# Single-nucleotide polymorphisms in *ialB*, *gltA* and *rpoB* genes of *Bartonella bacilliformis* isolated from patients in endemic Peruvian regions

**DOI:** 10.1371/journal.pntd.0011615

**Published:** 2023-10-10

**Authors:** Yanina Zarate-Sulca, Karen Daphne Calvay-Sanchez, Víctor Jimenez-Vasquez, Joaquim Ruiz, Oscar Acosta-Conchucos, Giovanna Mendoza-Mujica

**Affiliations:** 1 Laboratorio de Referencia Nacional de Metaxénicas y Zoonosis Bacterianas, Centro Nacional de Salud Pública, Instituto Nacional de Salud Perú, Lima, Perú; 2 Grupo de Investigación en Enfermedades Infecciosas Emergentes, Universidad Científica del Sur, Lima, Perú; 3 Facultad de Farmacia y Bioquímica, Universidad Nacional Mayor de San Marcos, Lima, Perú; University Hospital of Frankfurt, GERMANY

## Abstract

*Bartonella bacilliformis* is a Gram-negative, aerobic bacterium and the known causal agent of Carrion’s disease, still considered a neglected disease. There is limited information about the nucleotide sequences of this bacterium in international databases, and few studies have addressed the genetic diversity of *B*. *bacilliformis*. We analyzed a total of 20 isolates of *B*. *bacilliformis* from the Peruvian regions of Ancash and Cajamarca. Three genes (*ialB*, *gltA*, and *rpoB*) were sequenced in each isolate and nucleotide sequences retrieved from GenBank (16 *B*. *bacilliformis* genomes) were also included in the study. All this information was merged in order to obtain clearer evidence of the phylogenetic relationships of *B*. *bacilliformis*. In the phylogenetic analysis conducted with the concatenated markers, four isolates (B.b-1, B. b-3, B. b- 7, B.b-8) from the Ancash region were observed to form a subgroup different from *B*. *bacilliformis* type strain KC583, showing dissimilarity levels of 5.96% (*ialB*), 3.69% (*gltA*) and 3.04% (*rpoB*). Our results suggest that *B*. *bacilliformis* consists of two different subgroups. Future investigations are needed to establish the taxonomic status of these subgroups.

## Author summary

Bartonella bacilliformis is the causative agent of Carrion’s disease, which affects neglected communities of the South American Andes. The available information about the genomic aspect of B. bacilliformis is limited, and there is no information on the ecological and geographical bases of the divergence of this bacterium. In this study, three genes were sequenced in twenty isolates of B. bacilliformis from Ancash and Cajamarca, Peruvian endemic areas. The collected data suggest a specific subgroup of B. bacilliformis present in the Ancash region, it could therefore be assumed that there is a sub-speciation process in the strains of this endemic zone of Peru. Additionally, our findings show non-synonymous mutations that could trigger the alterations of the activity or structure of the proteins codified by the sequence genes.

## Introduction

*Bartonella bacilliformis* is a facultative intracellular pathogen and the causative agent of Carrion’s disease [1]. This pathogen is transmitted by sand flies (*Lutzomyia* spp.); therefore the distribution of the disease is associated with altitudes in which these arthropods are found [2]. Therefore, the distribution of this pathogen is restricted to the Andean valleys of Peru and Ecuador, [2,3]. While reported in Southern Colombia during the last century, no confirmed case has been reported in this country in the last few decades [4].

Carrion’s disease displays an acute phase manifesting with hemolytic anemia (named Oroya fever), which often precedes a chronic phase with skin lesions (named Peruvian Wart) [1]. Intense red blood cell destruction occurs in the acute phase, leading to severe hemolytic anemia and temporary immunosuppression [5,6]. The warts or eruptive lesions manifested in the chronic phase have several forms of presentation: miliary, mular, or nodular, with bleeding by the wart lesion being the most serious complication during this phase [4,6].

However, the biphasic manifestation is not reported in all patients. Thus, while some patients do not develop the verrucous phase after an acute episode, others begin with a verrucous episode without a previous report of an acute episode [4]. For instance, there is a predominance of the acute phase of the disease over the verrucous period in patients from the Monzón Valley in Huánuco and the Sacred Valley of the Incas in Cuzco [7].

The differences between these clinical pictures could be associated with the characteristics inherent to the circulating genotypes; hence this study aimed to determine the possible genetic differences in *B*. *bacilliformis* isolated from patients with Carrion’s disease in the Peruvian departments of Ancash and Cajamarca.

## Materials and methods

### Reactivation of cryopreserved strains

Twenty *B*. *bacilliformis* strains (10 from the Ancash department and 10 from the Cajamarca department) were reactivated in biphasic medium (RPMI Medium1640 with L-glutamine and bicarbonate (Gibco, Paisley, UK), and subcultured in Columbia agar medium (Columbia blood agar base; OXOID, Hampshire, UK) enriched at 10% with sheep blood. All the strains included in the study were isolated from patients for diagnostic purposes and provided by the Laboratorio de Referencia Nacional de Metaxénicas y Zoonosis Bacterianas of the Instituto Nacional de Salud, Peru. Additionally, the strain KC583 (ATCC 35685) was also reactivated to be used as a sequencing reference. The origin and year of the isolates are described in Table 1.

**Table 1:** General information on the *B*. *bacilliformis* strains included in the study.

Code	Place of Origin (Region, province)	Year
B. b-1	Ancash, Casma	2008
B. b-2	Ancash, ND	2015
B. b-3	Ancash, Casma	2008
B. b-4	Ancash, ND	2016
B. b-5	Ancash, ND	2016
B. b-6	Ancash, ND	2016
B. b-7	Ancash, ND	2008
B. b-8	Ancash, Casma	2008
B. b-9	Ancash, ND	2009
B. b-10	Ancash, Huaylas	2018
B. b-13	Cajamarca, Santa Cruz	2007
B. b-14	Cajamarca, ND	2018
B. b-15	Cajamarca, ND	2017
B. b-16	Cajamarca, ND	2015
B. b-17	Cajamarca, Chota	2009
B. b-18	Cajamarca, Santa Cruz	2009
B. b-19	Cajamarca, Santa Cruz	2007
B. b-20	Cajamarca, ND	2011
B. b-21	Cajamarca, San Ignacio	2019
B. b-22	Cajamarca, Chota	2010
KC583 (ATCC 35685)	Lima, Churcampa	1957

ND: No data available

### DNA extraction and amplification

Colonies were harvested and DNA was extracted using a commercial kit (GeneJet NGS Cleanup Kit; Thermo Fisher Scientific, Vilnius, Lithuania) following the manufacturer’s instructions. We employed previously designed oligonucleotides for the polymerase chain reaction (PCR) test of the three genes (Table 2).

**Table 2:** Oligonucleotide sequences used in polymerase chain reaction tests and sequencing.

Gene	Name	Sequence (5´-3´)	Size (base pairs)	References
*ialB*	*ialBF* *ialBR*	AAGGAACTGGATGTAAAAT CTAAAAAGCACTCAAAAGAC	570	Padilla *et al*. *2003*[8]
*gltA*	BHCS 781p (F) BHCS 1137n (R)	GGGGACCAGCTCATGGTGG AATGCAAAAAGAACAGTAAACA	338	Noman *et al*. 1995[9]
*rpoB*	*rpoB*1435F *rpoB*2327R	CGCATTGGTTTACTTCGTATG GTAGACTGATTAGAACGCTG	589	Mullins *et al*. 2013[10]

The reactions were carried out in 40 μl of master mix containing 4.5 μL of PCR buffer 10X, 2.5 mM of MgCl_2_, 0.25 mM of dNTPs, 0.25 mM of each primer, 0.75 U/ μL of Platinum T*aq* DNA Polymerase (Thermo Fisher Scientific, Sao Paulo, Brazil), with at least 2 ng/μL of DNA template. The amplification protocol for *ialB* and *gltA* genes proceeded with 40 cycles at 94°C for 30 sec, 55°C for 30 sec, and 72°C for 1 min. *rpoB* gene amplification was performed by 45 cycles at 95°C for 30 sec, 51°C for 30 sec, and 72°C for 1 min. PCR products were visualized on a 1.6% agarose gel with nucleic acid gel stain (Syber gold; Life Technologies, Oregon, USA).

### Sequencing reaction

The PCR products were purified using a commercial kit (GeneJet NGS Cleanup Kit; Thermo Fisher Scientific) according to the specifications of the manufacturer, and the purified products were processed using a commercial kit (BigDye Terminator v3.1 Cycle Sequencing kit; Thermo Fisher Scientific). The sequencing reactions were done using 35 cycles with the following conditions: denaturation at 96°C for 10 sec, hybridization at 50°C for 45 sec, and extension at 60°C for 60 sec. The treated products were purified using *in-house* precipitation buffer; thus, each sample was treated using 3μL of 3M sodium acetate pH 5.4, 62.5 μL of absolute ethanol, and 14.5 μL of molecular grade water.

After mixing with precipitation buffer, the products were incubated at room temperature for 30 min and thereafter centrifuged at 2000g for 30 min. The pellets were resuspended in 90 μL of 70% ethyl alcohol, and then were centrifuged at 2000g for 15 min. 10 μL of deionized formamide was added to the dried pellet, and samples were denatured at 96°C for 2 min followed by fast cooling on ice. The DNA sequencing was performed using an Applied Biosystems Genetic Analyzer (3500xL, Thermo Fisher Scientific, United States).

### Gene data analysis

Sixteen sequences from *B*. *bacilliformis* genomes and four sequences from genomes of other *Bartonella* spp. available in the NCBI GenBank database were included for multiple alignments and the elaboration of the phylogenetic tree (Table 3). In addition, the analysis of *gltA* included sequences previously reported by Birtles *et al*. (strain LA6.3) [11] and Lydy *et al*. (strain Vega) [12].

**Table 3:** Genomes retrieved from GenBank.

Strain	Accession number	Origin	Year	Authors
Ver097	NZ_KL503803.1	Caraz, Ancash	1997	Feldgarden,M., *et al*.
KC583 (ATCC 35685)	NC_008783.1	Huarochiri, Lima	1957	Hendrix,L., *et al*.
KC584	NZ_CP045671.1	Churcampa, Huancavelica	1959	Dichter,A., *et al*.*
USM-LMMB 07	NZ_LQXX01000004.1	Carmen de la Frontera, Piura	2011	Guillen,Y., *et al*. **
USM-LMMB 06	NZ_LQWW01000008.1	La Convención (Quillabamba), Cusco	2011	Guillen,Y., *et al*.
Cond044	NZ_KL503800.1	Huaylas, Ancash	1993	Feldgarden,M., *et al*.
Ver075	NZ_KL503823.1	Caraz, Ancash	1997	Feldgarden,M., *et al*.
San Pedro600-02	NZ_KK097680.1	Caraz, Ancash	1997	Feldgarden,M., *et al*.
Hosp800-02	NZ_KL503780.1	Caraz, Ancash	1997	Feldgarden,M., *et al*.
CAR600-02	NZ_KL503827.1	Caraz, Ancash	1905	Feldgarden,M., *et al*.
Heidi Mejia	NZ_KK097685.1	Caraz, Ancash	1997	Feldgarden,M., *et al*.
Peru-18	NZ_KK097689.1	Ollantaytambo, Cusco	1998	Feldgarden,M., *et al*.
Peru38	NZ_KL503811.1	Calca, Cusco	1905	Feldgarden,M., *et al*.
VAB9028	NZ_KL503817.1	Tingua, Ancash	1984	Feldgarden,M., *et al*.
CUSCO5	NZ_KL503796.1	Urubamba, Cusco	1998	Feldgarden,M. *et al*.
INS	NZ_AMQK01000005.1	Jaen, Cajamarca	2010	Tarazona,D., *et al*. ***

Data extracted from references, GenBank sequences as well as the webpage https://pubmlst.org/organisms/bartonella-bacilliformis.

All strains were isolated in Peru

*Reference[14], **[15], *** Reference [16]

The quality and assembly of the sequences were determined using the free program Seq Trace (https://bio.tools/seqtrace), which evaluated the reading of each base. To determine the identity of the sequences obtained at the genus and species level, the sequences were BLAST searched against the sequences deposited in the NCBI GenBank database (https://blast.ncbi.nlm.nih.gov/Blast.cgi). The Weblogo server was employed for easy visualization of amino acid multiple sequence alignment, with the overall height of the stack indicating the sequence conservation at that position, while the height of symbols within the stack indicates the relative frequency of each amino or nucleic acid at that position [13].

The multiple alignments were performed using CLC Genomics Workbench 21.0.5 and MEGA 7.0.26. The phylogenetic analysis was carried out using Neighbor Joining (NJ) based on genetic distances using MEGA X, a nucleotide substitution model estimated with the JModeltest 2 program, pairwise deletion option and 1000 bootstrap repetitions.

In all cases, the sequences (DNA and amino acid) were compared to those of the reference strain KC583 (ATCC35685; NC_008783.1).

## Results

### Bioinformatic analysis of the *ialB* region: a gene associated with red blood cell invasion

**Fig 1:**
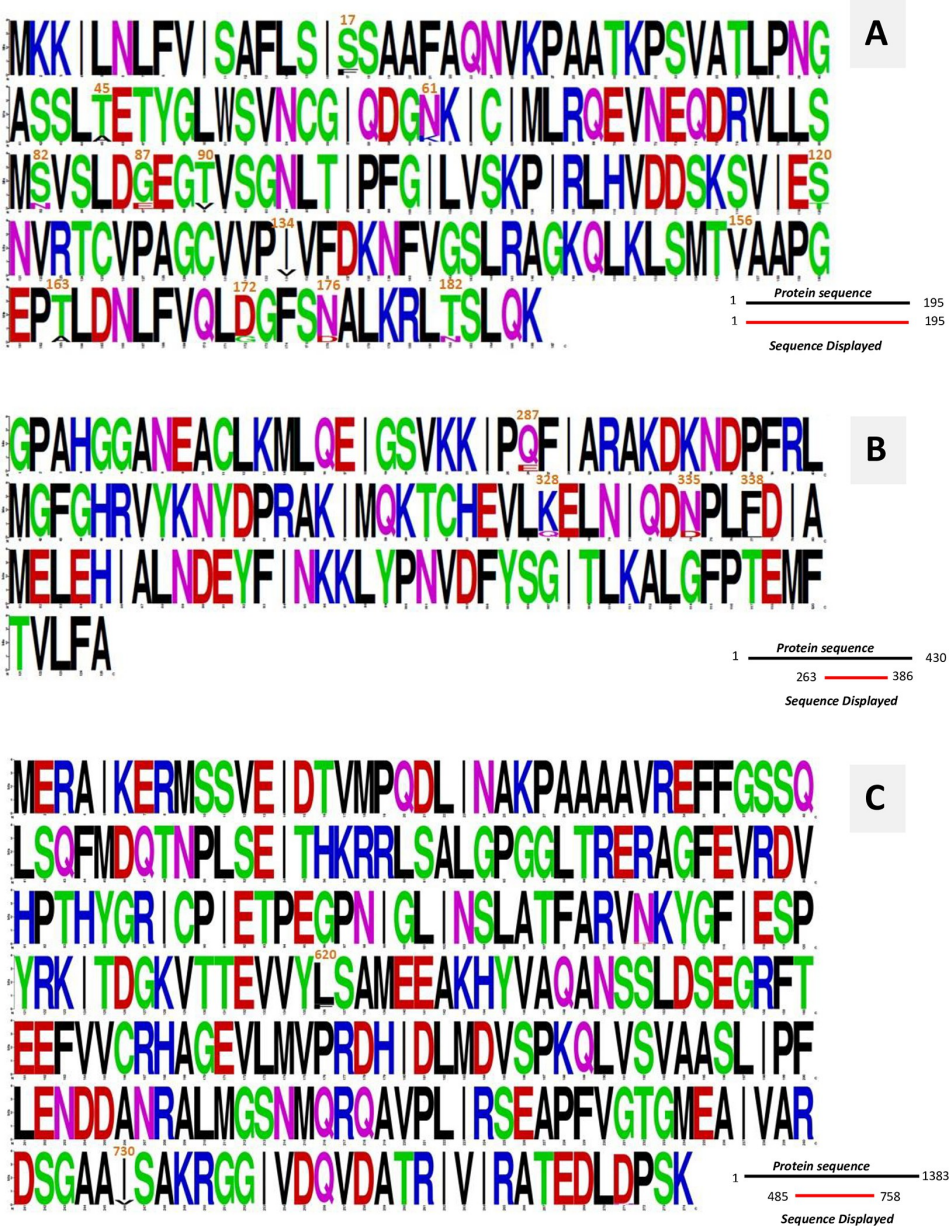
Sequence logo of the multiple sequence alignment of amino acid sequence for the identification of non-synonymous mutations: A) IalB; B) GltA; C) RpoB. Amino acids are colored according to their chemical properties: polar amino acids are green, basic amino acids are blue, acidic amino acids are red, and hydrophobic amino acids are black.

In the analysis of the *ialB* gene, four *B*. *bacilliformis* strains (B.b-1, B. b-3, B. b- 7, B.b-8) had an identity percentage of 94.04% with *B*. *bacilliformis* KC583 (ATCC 35685, CP000524.1). These four strains isolated from Ancash displayed 31 nucleotide variations (Table 4, S1A Fig), which lead to 13 predicted non-synonymous amino acid substitutions (Table 5; Fig 1). The DNA sequences of these 4 isolates were 100% identical to the ialB sequence of *B*. *bacilliformis* Ver097 (NZ_KL503803.1) (identity percentage, 100%). In the multiple-sequence alignment of the remaining 16 strains, 10 from Cajamarca and six from Ancash, no variations were detected in comparison to the ATCC 35685 reference strain (S1 Fig).

**Table 4:** Single nucleotide variations shown across all three sequenced regions (*ialB*, *gltA* and *rpoB*).

**Strain**	Position of nucleotide variations in *ialB* gene (1-561)*															
	**50**	**60**	**108**	**133**	**138**	**183**	**186**	**234**	**245**	**260**	**267**	**268**	**269**	**279**	**304**	**312**
**KC583**	C	G	A	A	G	C	A	A	G	G	T	A	C	C	T	T
**B.b-1**	T	A	G	G	A	A	G	G	A	A	G	G	T	A	C	C
**B.b-3**	T	A	G	G	A	A	G	G	A	A	G	G	T	A	C	C
**B.b-7**	T	A	G	G	A	A	G	G	A	A	G	G	T	A	C	C
**B.b-8**	T	A	G	G	A	A	G	G	A	A	G	G	T	A	C	C
**Ver097**	T	A	G	G	A	A	G	G	A	A	G	G	T	A	C	C
	**327**	**342**	**359**	**400**	**432**	**441**	**448**	**466**	**487**	**489**	**501**	**515**	**526**	**545**	**560**	
**KC583**	G	G	G	A	A	C	T	G	**A**	C	A	A	A	C	A	
**B.b-1**	A	A	C	G	G	T	C	A	G	T	G	G	G	A	G	
**B.b-3**	A	A	C	G	G	T	C	A	G	T	G	G	G	A	G	
**B.b-7**	A	A	C	G	G	T	C	A	G	T	G	G	G	A	G	
**B.b-8**	A	A	C	G	G	T	C	A	G	T	G	G	G	A	G	
**Ver097**	A	A	C	G	G	T	C	A	G	T	G	G	G	A	G	
**Strain**	Position of nucleotide variations in *gltA* gene (782-1160)*															
	**786**	**834**	**840**	**859**	**951**	**982**	**1003**	**1012**	**1035**	**1065**	**1077**	**1095**	**1134**	**1155**		
**KC583**	G	G	C	C	G	A	A	T	G	T	C	C	G	C		
**B.b-1**	A	A	T	G	A	C	G	C	A	C	T	T	C	T		
**B.b-3**	A	A	T	G	A	C	G	C	A	C	T	T	C	T		
**B.b-7**	A	A	T	G	A	C	G	C	A	C	T	T	C	T		
**B.b-8**	A	A	T	G	A	C	G	C	A	C	T	T	C	T		
**Ver097**	A	A	T	G	A	C	G	C	A	C	T	T	C	T		
**Strain**	Position of nucleotide variations in *rpoB* gene (1451-2275)*															
	**1464**	**1473**	**1539**	**1551**	**1554**	**1563**	**1644**	**1686**	**1701**	**1722**	**1731**	**1752**	**1770**	**1806**	**1809**	**1827**
**KC583**	G	A	G	C	G	G	G	C	G	G	G	A	G	G	T	C
**B.b-1**	C	G	A	T	A	A	A	T	A	T	A	G	A	A	**C**	T
**B.b-3**	C	G	A	T	A	A	A	T	A	T	A	G	A	A	**C**	T
**B.b-7**	C	G	A	T	A	A	A	T	A	T	A	G	A	A	**C**	T
**Ver097**	C	G	A	T	A	A	A	T	A	T	A	G	A	A	**C**	T
**KC583**	**1839**	**1842**	**1858**	**1896**	**2109**	**2118**	**2121**	**2157**	**2188**	**2274**						
**B.b-1**	**G**	**G**	C	A	A	G	C	G	A	A						
**B.b-3**	A	A	T	G	G	A	T	A	G	G						
**B.b-7**	A	A	T	G	G	A	T	A	G	G						
**Ver097**	A	A	T	G	G	A	T	A	G	G						

*Sequenced region

**Table 5:** Predicted amino acid substitutions in *B*. *bacilliformis* presenting DNA differences compared to the *B*. *bacilliformis* reference strain KC583 (ATCC35685).

Positions*	*ialB*													*gltA*				*rpoB*	
	17	45	61	82	87	90	120	134	156	163	172	176	182	287	328	335	338	620	730
**KC583**	**S**	**T**	**N**	**S**	**G**	**T**	**S**	**I**	**V**	**T**	**D**	**N**	**T**	**Q**	**K**	**N**	**F**	**L**	**I**
**Ver097**	F	A	K	A	E	V	T	V	I	A	G	D	N	E	Q	D	L	F	V
**B.b.-1**	F	A	K	A	E	V	T	V	I	A	G	D	N	E	Q	D	L	F	V
**B.b.-3**	F	A	K	A	E	V	T	V	I	A	G	D	N	E	Q	D	L	F	V
**B.b.-7**	F	A	K	A	E	V	T	V	I	A	G	D	N	E	Q	D	L	F	V
**B.b.-8**	F	A	K	A	E	V	T	V	I	A	G	D	N	E	Q	D	L		

* Amino acid positions

### Bioinformatic analysis of the *glt*A gene: citrate synthase

Fourteen single-nucleotide variations were identified in the *glt*A gene of the *B*. *bacilliformis* isolates B.b.-1, B.b -3, B.b.-7 y B.b.-8 (Table 4, S1B Fig); these mutations resulted in 4 predicted amino acid substitutions (Table 5, Fig 1B). As occurred with the *ialB* gene, the variations were identical to the isolate Ver097 and to the two non-genome *gltA* sequences obtained from GenBank (Accession number: DQ200879.1; Z70021.1). The other data retrieved from the GenBank did not present any nucleotide variation with respect to the reference sequence.

### Bioinformatic analysis of the *rpoB* gene: β subunit of RNA polymerase

In the bioinformatic analysis of the nucleotide sequences of the *rpoB* gene, 26 single-nucleotide polymorphisms were identified leading to 2 amino acid substitutions (Tables 4 and 5) as in the *ialB* and *gltA* genes, in the same four strains from the Ancash region obtained in 2008 (B.b.-1, B.b.-3, B.b.-7 and B.b.-8), and as above, the substitutions were identical to those present in the Ver097 strain. The Cond044 strain presented 6 nucleotide variations and only 1 amino acid substitution 755 (Asp—> Asn). Likewise, strain Ver075 presented a single nucleotide variation which would not generate amino acid substitution. The remaining samples and retrieved data did not present any nucleotide variation relative to strain KC583. Changes were identified in the Cond044 sequence at positions 1752, 1806, 1878, 1896, 2109 and 2263 as well as in the Ver075 sequence at position 1786.

To sum up, four Ancash-isolates displayed amino acid substitutions in the three predicted proteins (IalB, GltA and RpoB) in comparison to the reference strain KC583. However, the amino acid substitutions were identical to the amino acids predicted in theVer097 strain (Table 5).

### Phylogenetic analysis based on three gene markers

**Fig 2:**
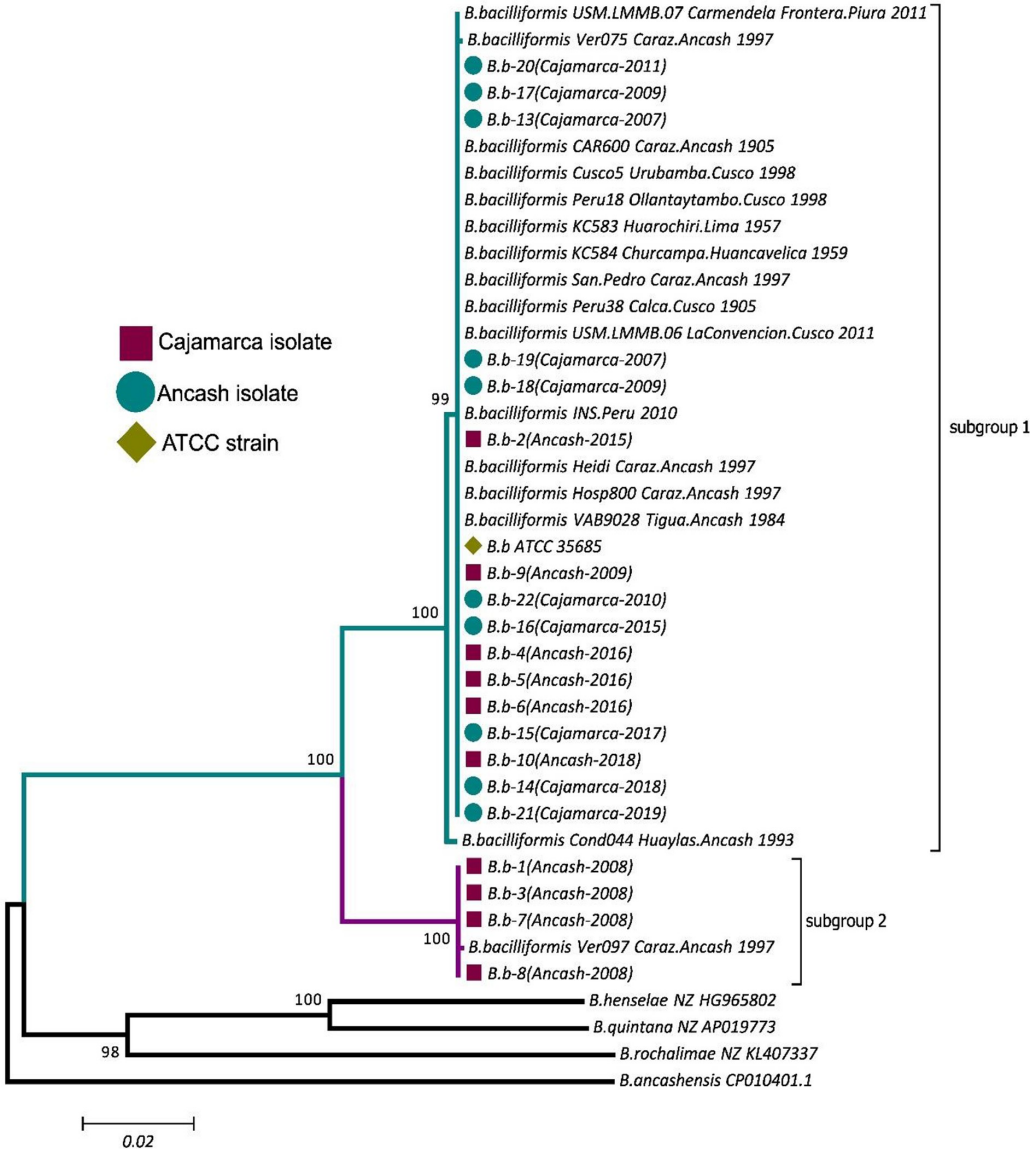
Concatenated phylogenetic tree based on the *ialB*, *gltA*, *rpoB genes* from 20 strains isolated from Cajamarca and Ancash, and 20 genomes downloaded from GenBank. For the construction of the tree, the NJ method with 1,000 replicates was used. The Bootstrap values are displayed between the branches respectively. The tree was made in the MEGA 7.0 program.

In all cases, phylogenetic analysis of the three analyzed genes presented two main groups. One of them was made up of the same four strains B.b-1, B.b-3, B.b-7, B.b-8, while the 16 remaining strains were grouped together with the KC583 reference strain. The inclusion of the sequences of *ialB*, *gltA* and *rpoB* extracted from *B*. *bacilliformis* genomes present in GenBank, showed that all of them except those from Ver097 were almost identical to that of KC583, with Cond044 and Ver075 showing minor differences in *rpoB* as described above (S4 Fig). The *gltA* gene of strains Vega (GenBank access: DQ200879.1), and LA6.3 (GenBank access: Z70021.1) group together with Ver097 and the isolates B.b-1, B. b-3, B. b-7, B. b-8. (S3 Fig). Subsequently, this also was the scenario shown when a phylogenetic tree based on the concatenated *ialB*, *gltA*, and *rpoB* genes was generated (Fig 2).

## Discussion

The *Bartonella* genus currently comprises 38 species with standing in nomenclature and an undefined number of *Candidatus* species, including uncultured isolates for which only partial DNA sequences have been obtained as well as isolates from which whole genomic data are available (https://lpsn.dsmz.de/search?word=bartonella). The DNA fragment most widely used to study the taxonomy of the *Bartonella* genus is 16S rDNA, but it is not recommended for speciation studies [17]. Hence, the *gltA* and *rpoB* genes and 16S-23S rDNA ITS were used to classify *Bartonella* species [18,19]. In relation to *B*. *bacilliformis*, the detection of the *ialB* gene has been recommended for identification purposes since it was considered to be an exclusive gene of this bacterium [8]. However, further studies showed the presence of the *ialB* gene in other species of the genus and other genera, and this application was subsequently dismissed [20]. In the present study, nucleotide sequences of three genes (*ialB*, *gltA*, and *rpoB*) of 20 isolates of *B*. *bacilliformis* from Ancash and Cajamarca were obtained, and 16 sequences of *B*. *bacilliformis* were retrieved from GenBank. These data were used to obtain clearer evidence of the phylogenetic relationship of *B*. *bacilliformis*. Additionally, this analysis helps to drive future investigations since this study is an initial screening with a cost-effective approach, which will allow the selection of *B*. *bacilliformis* strains for further whole genome sequencing.

To the best of knowledge, there are no data related to single-nucleotide polymorphisms in the *ialB* gene of *B*. *bacilliformis*. In the present study, in four of the 20 isolates, 16 synonymous mutations and 15 non-synonymous mutations were detected which resulted in 13 amino acid substitutions (Fig 1A). These amino acid substitutions were identified in four *B*. *bacilliformis* strains of Ancash, a Peruvian department in which the main clinical form of Carrions disease is the eruptive or verrucous phase [21]. According to Coleman and Minnick, the disruption of the *ialB* gene of *B*. *bacilliformis* resulted in a 47% to 53% decrease in erythrocytes association compared to the wild-type, parental strain [22]. Similarly, it has been reported that a inactivated *ialB* gene from *Bartonella tribocorum* or *Bartonella birtlesii* did not generate bacteremia or invasion in rat or mouse erythrocytes in *in vivo* tests [23,24]. Hence, it could be inferred that the samples that present amino acid substitutions might have an altered affinity to red blood and epithelial cells because amino acid substitutions could generate structural and functional changes in the invasion protein, likewise, we assume amino acid substitutions could affect not only infection in the host or reservoir but also the clinical manifestations of the infection by *B*. *bacilliformis*. Additionally, according to Coleman *et al*., environmental changes (transition from sandfly to humans) could influence the expression of the *ialB* gene [22]. Correlation studies determined that the highest expression of the *ialB* gene was in *B*. *bacilliformis* strains treated with temperatures of 20°C [25]. While further studies are necessary, several of the amino acid substitutions observed might have an impact on protein functionality as they result in changes from positively charged to other negatively charged amino acids (or vice versa).

Analysis of the *gltA* gene showed single-nucleotide mutations in the same four strains that contain mutations in the *ialB* gene and a degree of dissimilarity of 3.68%, with the sequences being identical to those of the Ver097, LA6.2 and Vega isolates. This result agrees with that obtained by Birtles *et a*l. who analyzed a partial sequence of *gltA* in *B*. *bacilliformis*; while they showed that this sequence was highly conserved, the *gltA* sequences of isolates LA6.2, LA6.3 and Luc-Uba presented 3% dissimilarity with respect to other *B*. *bacilliformis* [11]. Currently, the *gltA* gene is used to classify species or subgroups of *Bartonella spp*. Thus, previous studies comparing partial sequences of the *gltA* gene of *Bartonella spp*. found 83.8% - 93.5% similarity between different *Bartonella* species and 99.6–99.8% similarity of isolates belonging to the same species [18]. To the best of our knowledge, there are no studies related to mutations in the *gltA* gene of *B*. *bacilliformis* and the effect that these could have on GltA catalytic activity.

It should be noted that previous studies about multi-locus sequence typing (MLST) studies did not include the *ialB* and *gltA* genes, but the present study confirms that the markers chosen have discriminatory power for discriminating between *B*. *bacilliformis* subgroups (S2 and S3, Fig 2).

Regarding the *rpoB* gene (Figs 1C and S1C), three out of four isolates presenting substitutions in the *ialB* and *gltA* genes also presented 26 nucleotide variations leading to two non-synonymous mutations. The *rpoB* gene of the remaining isolate (B.b-8) could not be amplified, the reason could not be determined but might be due to a polymorphism in the primer annealing region which prevents annealing and subsequent amplification. The results of the analysis had a coverage of 100% and a dissimilarity of 3.04% with respect the reference strain KC583. This latter result is in agreement with that obtained by other researchers who have reported a 3.0% dissimilarity in the *rpoB* gene during sequence comparison in a MLST analysis [26]. It is important to point out that amino acid substitutions at RpoB can generate resistance to rifampicin in different microorganisms, including *Mycobacterium tuberculosis* [27]. Regarding *B*. *bacilliformis* the presence of the amino acid substitutions at positions 527 (Gln—> Arg), 531 (Ser—> Phe), 540 (His—> Tyr), 545 (Ser—> Phe), and 588 (Ser—> Tyr) have been shown to be involved in the development of resistance to rifampicin [28,29]. Nevertheless, amino acid positions 620 and 730 of *B*. *bacilliformis* have not been related to the development of rifampicin resistance. Furthermore they are outside the equivalent RpoB regions classically involved in this resistance and therefore probably represent polymorphisms [30].

Analyzing the ITS region, Houpikian *et al*. reported variations of dissimilarity between three subspecies of *B*. *vinsonii* [31] and suggested that these subgroups could belong to a different taxon within the species. In this study, the isolates B.b.-1, B.b.-3, B.b.-7 and B.b.-8 consistently presented dissimilarities >3% in all the genes analyzed when compared with the strain KC583, but a 100% identity with those of strain Ver097. Other studies have shown similar scenarios analyzing other *B*. *bacilliformis* genes, as for instance Pap31, with a 10.3% of dissimilarity between KC583 and Ver097 [32] and these differences are also reflected in multi-locus sequence typing analysis (MLST), with the strains Ver097, LA6.3, Luc-Uba as outliers [33]. This scenario allows us to suggest that they belong to at least to a *B*. *bacilliformis* subspecies, This possibility was previously reported by Paul *et al* [34] who suggested that isolates Ver097, Luc-Uba and LA6.3 make up a *B*. *bacilliformis* subspecies.

Currently, there is no information on the ecological and geographical bases of the divergence in *B*. *bacilliformis*, however, we identified that there is a specific subgroup of *B*. *bacilliformis* present in the Ancash region. It could therefore be assumed that there is a sub-speciation process in the strains of this endemic zone of Peru. This statement is supported by Paul *et al*. who described potential sub-speciation in isolates related to Ver097, such as, for instance, LA6.3, and Luc-Uba, all three of which were isolated from Ancash [34]. As mentioned, our findings show amino acid mutations that could affects protein functions, so we consider these non-synonymous mutations must be carefully analyzed to avoid inaccuracy during an *in-silico* analysis [35].

Nevertheless, there is a limited number of isolates, and further studies with a higher number of samples from Ancash and other regions are required for final conclusions to be made, especially if differences in clinical manifestations are identified in patients from Ancash where the eruptive phase is more predominant in comparison to other endemic areas, such as Cajamarca, Cusco and Huánuco [18]. A limitation of the present study is that it was based on the analysis of 3 relevant genes, and that larger data sets from whole genome sequencing will result in a more in-depth analysis. Nevertheless, the obtained results clearly highlight the presence of in-depth differences between both groups of *B*. *bacilliformis* included in the analysis.

## Conclusion

The analysis of the single-nucleotide polymorphisms of the *gltA*, *ialB* and *rpoB* genes is supported by other investigations, providing more evidence of the genotypic diversity of *B*. *bacilliformis* circulating in Peru. However, this research needs to be reinforced by complete analysis of whole genome sequencing in a greater number of isolates from different regions of Peru.

## Supporting information

S1 Fig Single-nucleotide variations displayed along the aligned sequences of: A)*ial*B; B) *gltA*, consensus sequence includes only 782–1160 nucleotide sequences; C) *rpoB*, consensus sequence includes only 1451–2275 nucleotide sequences.
(PDF)

S2 Fig Phylogenetic tree based on the *ialB* gene in 10 isolates from Cajamarca and 10 from Ancash and 17 genomes downloaded from the Genbank. The construction of the tree used the NJ method with 1,000 replicates. The Bootstrap values are displayed between the branches respectively. The tree was made in the MEGA 7.0 program. Red and green symbols highlight strains sequenced in the study.
(PDF)

S3 Fig Phylogenetic tree based on the *gltA* gene of 10 *B*. *bacilliformis* isolated in Cajamarca and 10 from Ancash; 17 genomes and 2 partial genes downloaded from the GenBank. The construction of the tree used the NJ method with 1,000 replicates. Bootstrap values are shown between branches respectively. The tree was made in the MEGA 7.0 program. Red and green symbols highlight strains sequenced in the study.
(PDF)

S4 Fig Phylogenetic tree based on the *rpoB gene* from 10 *B*. *bacilliformis* isolates from Cajamarca and 10 from Ancash, and 17 genomes downloaded from the GenBank. For the construction of the tree, the NJ method with 1,000 replicates was used. Bootstrap values are shown between branches and evolutionary distances are calculated using Kimura’s 2-parameter method. The tree was made in the MEGA 7.0 program. Red and green symbols highlight strains sequenced in the study.
(PDF)

## References

[1] Harms A, Dehio C. Intruders below the radar: molecular pathogenesis of *Bartonella spp*. Clin Microbiol Rev. 2012;25(1):42–78. 22232371 10.1128/CMR.05009-11 PMC3255967

[2] Maguiña Vargas C, Ugarte-Gil C, Breña Chávez P, Ordaya Espinoza E, Ventosilla López P, Huarcaya Castilla E, et al. Actualización de la enfermedad de Carrión. Rev Medica Hered. 2008;19(1): 36–41.

[3] Minnick MF, Anderson BE, Lima A, Battisti JM, Lawyer PG, Birtles RJ. Oroya fever and verruga peruana: bartonelloses unique to South America. PLoS Negl Trop Dis. 2014;8(7):e2919. doi: 10.1371/journal.pntd.0002919 25032975 PMC4102455

[4] Ruiz J. JMM Profile: *Bartonella bacilliformis*: a forgotten killer. J Med Microbiol. 2022;71(12):001614. 10.1099/jmm.0.001614 36748511

[5] Ticona E, Huaroto L, Garcia Y, Vargas L, Madariaga MG. The pathophysiology of the acute phase of human bartonellosis resembles AIDS. Med Hypotheses. 2010;74(1):45–9. doi: 10.1016/j.mehy.2009.06.054 19665314

[6] Pachas Chavez PE. Epidemiologia de la bartonelosis en el Perú. Módulos Técnicos Serie Documentos Monográficos.Oficina General de Epidemiología / Instituto Nacional de Salud. Lima, Perú; 2000.

[7] Tejada Valencia AI, Vizcarra H, Pérez J, Cáceres L. AG, Quispe J, Pinto J, et al. Estudio clínico epidemiológico de bartonelosis humana en el valle del Monzón, Humalíes, Huánuco. Fac Med Perú. 2003;64(4)211–7.

[8] Padilla R C, Ventura E G. Diseño y estandarización de una prueba de PCR para el diagnóstico de la Bartonelosis causada por *Bartonella bacilliformis*. Rev Peru Med Exp Salud Publica. 2003;20(1): 5–8.

[9] Norman AF, Regnery R, Jameson P, Greene C, Krause DC. Differentiation of Bartonella-like isolates at the species level by PCR-restriction fragment length polymorphism in the citrate synthase gene. J Clin Microbiol. 1995;33(7): 1797–803. doi: 10.1128/jcm.33.7.1797-1803.1995 7545181 PMC228273

[10] Mullins KE, Hang J, Jiang J, Leguia M, Kasper MR, Maguiña C, et al. Molecular Typing of “*Candidatus* Bartonella ancashi,” a New Human Pathogen Causing Verruga Peruana. J Clin Microbiol. 2013;51(11): 3865–8. 23985925 10.1128/JCM.01226-13 PMC3889784

[11] Birtles RJ, Fry NK, Ventosilla P, Cáceres AG, Sánchez E, Vizcarra H, et al. Identification of *Bartonella bacilliformis* genotypes and their relevance to epidemiological investigations of human bartonellosis. J Clin Microbiol. 2002;40(10): 3606–12. 12354853 10.1128/JCM.40.10.3606-3612.2002 PMC130851

[12] Lydy SL, Eremeeva ME, Asnis D, Paddock CD, Nicholson WL, Silverman DJ, et al. Isolation and characterization of *Bartonella bacilliformis* from an expatriate Ecuadorian. J Clin Microbiol. 2008;46(2): 627–37. 18094131 10.1128/JCM.01207-07 PMC2238110

[13] Crooks GE, Hon G, Chandonia JM, Brenner SE. WebLogo: a sequence logo generator. Genome Res. 2004;14(6): 1188–90. doi: 10.1101/gr.849004 15173120 PMC419797

[14] Dichter AA, Schultze TG, Becker SA, Tsukayama P, Kempf VAJ. Complete Genome Sequence of *Bartonella bacilliformis* Strain KC584 (ATCC 35686). Microbiol Resour Announc. 2020;9(1): e01377–19. 31896649 10.1128/MRA.01377-19 PMC6940301

[15] Guillen Y, Casadellà M, García-de-la-Guarda R, Espinoza-Culupú A, Paredes R, Ruiz J, et al. Whole-Genome Sequencing of Two *Bartonella bacilliformis* Strains. Genome Announc. 2016;4(4): e00659–16. 27389274 10.1128/genomeA.00659-16 PMC4939791

[16] Tarazona D, Padilla C, Cáceres O, Montenegro JD, Bailón H, Ventura G, et al. Whole Genome Sequencing and Comparative Analysis of *Bartonella bacilliformis* Strain INS, the Causative Agent of Carrion’s Disease. Genome Announc. 2013;1(1): e00053–12. 10.1128/genomeA.00053-12 PMC3569277 23409255

[17] Fox GE, Wisotzkey JD, Jurtshuk P. How close is close: 16S rRNA sequence identity may not be sufficient to guarantee species identity. Int J Syst Bacteriol. 1992;42(1): 166–70. doi: 10.1099/00207713-42-1-166 1371061

[18] Birtles RJ, Raoult D. Comparison of partial citrate synthase gene (gltA) sequences for phylogenetic analysis of *Bartonella species*. Int J Syst Bacteriol. 1996;46(4): 891–7. 8863415 10.1099/00207713-46-4-891

[19] Renesto P, Gouvernet J, Drancourt M, Roux V, Raoult D. Use of rpoB Gene Analysis for Detection and Identification of *Bartonella species*. J Clin Microbiol. 2001;39(2): 430–7. 11158086 10.1128/JCM.39.2.430-437.2001 PMC87755

[20] Deng H, Pang Q, Xia H, Le Rhun D, Le Naour E, Yang C, et al. Identification and functional analysis of invasion associated locus B (IalB) in *Bartonella species*. Microb Pathog. 2016;98: 171–7. 27515099 10.1016/j.micpath.2016.05.007

[21] Pachas Chavez PE. Enfermedad de Carrión (Bartonelosis) en el Perú. Lima: Ministerio de Salud, Oficina General de Epidemiología: Instituto Nacional de Salud; 2001. 88 p.

[22] Coleman SA, Minnick MF. Establishing a direct role for the Bartonella bacilliformis invasion-associated locus B (IalB) protein in human erythrocyte parasitism. Infect Immun. 2001;69(7): 4373–81. doi: 10.1128/IAI.69.7.4373-4381.2001 11401976 PMC98509

[23] Saenz HL, Engel P, Stoeckli MC, Lanz C, Raddatz G, Vayssier-Taussat M, et al. Genomic analysis of *Bartonella* identifies type IV secretion systems as host adaptability factors. Nat Genet. 2007;39(12): 1469–76. 18037886 10.1038/ng.2007.38

[24] Vayssier-Taussat M, Le Rhun D, Deng HK, Biville F, Cescau S, Danchin A, et al. The Trw type IV secretion system of *Bartonella* mediates host-specific adhesion to erythrocytes. PLoS Pathog. 2010;6(6): e1000946. 20548954 10.1371/journal.ppat.1000946 PMC2883598

[25] Coleman SA, Minnick MF. Differential expression of the invasion-associated locus B (*ialB*) gene of *Bartonella bacilliformis* in response to environmental cues. Microb Pathog. 2003;34(4): 179–86. 12668141 10.1016/s0882-4010(03)00005-6 PMC4104752

[26] Chaloner GL, Palmira Ventosilla, Birtles RJ. Multi-Locus Sequence Analysis Reveals Profound Genetic Diversity among Isolates of the Human Pathogen *Bartonella bacilliformis*. PLoS Negl Trop Dis. 2011;5(7): e1248. 21811647 10.1371/journal.pntd.0001248 PMC3139668

[27] Yam WC, Tam CM, Leung CC, Tong HL, Chan KH, Leung ETY, et al. Direct detection of rifampin-resistant Mycobacterium tuberculosis in respiratory specimens by PCR-DNA sequencing. J Clin Microbiol. 2004;42(10): 4438–43. doi: 10.1128/JCM.42.10.4438-4443.2004 15472290 PMC522342

[28] Gomes C, Martínez-Puchol S, Ruiz-Roldán L, Pons MJ, Del Valle Mendoza J, Ruiz J. Development and characterisation of highly antibiotic resistant *Bartonella bacilliformis* mutants. Sci Rep. 2016;6: 33584. 27667026 10.1038/srep33584 PMC5035977

[29] Biswas S, Raoult D, Rolain JM. Molecular mechanisms of resistance to antibiotics in *Bartonella bacilliformis*. J Antimicrob Chemother. 2007;59(6): 1065–70. 17449882 10.1093/jac/dkm105

[30] Severinov K, Soushko M, Goldfarb A, Nikiforov V. Rifampicin region revisited. New rifampicin-resistant and streptolydigin-resistant mutants in the beta subunit of *Escherichia coli* RNA polymerase. J Biol Chem. 1993;268(20): 14820–5. 8325861

[31] Houpikian P, Raoult D. 16S/23S rRNA intergenic spacer regions for phylogenetic analysis, identification, and subtyping of *Bartonella species*. J Clin Microbiol. 2001;39(8): 2768–78. 11473990 10.1128/JCM.39.8.2768-2778.2001 PMC88237

[32] Ruiz J, Gomes C. In silico analysis of Pap31 from *Bartonella bacilliformis* and other *Bartonella spp*. Infect Genet Evol J Mol Epidemiol Evol Genet Infect Dis. 2020;84: 104482. 10.1016/j.meegid.2020.104482 32738366

[33] Ruiz J, Pons MJ. Revisiting *Bartonella bacilliformis* MLST. Infect Genet Evol J Mol Epidemiol Evol Genet Infect Dis. 2018;63: 231–5. 10.1016/j.meegid.2018.05.032 29864510

[34] Paul S, Minnick MF, Chattopadhyay S. Mutation-Driven Divergence and Convergence Indicate Adaptive Evolution of the Intracellular Human-Restricted Pathogen, *Bartonella bacilliformis*. PLoS Negl Trop Dis. 2016;10(5): e0004712. 27167125 10.1371/journal.pntd.0004712 PMC4864206

[35] Jimenez-Vasquez V, Calvay-Sanchez KD, Zarate-Sulca Y, Mendoza-Mujica G. In-silico identification of linear B-cell epitopes in specific proteins of *Bartonella bacilliformis* for the serological diagnosis of Carrion’s disease. PLoS Negl Trop Dis. 2023;17(5): e0011321. 37228134 10.1371/journal.pntd.0011321 PMC10246785

