# Supplementary material for: Single-nucleotide polymorphisms in ialB, gltA and rpoB genes of Bartonella bacilliformis isolated from patients in endemic Peruvian regions
Source: PLoS Negl Trop Dis. 2023 Oct 10;17(10):e0011615. doi: 10.1371/journal.pntd.0011615 (PMC10564245; doi:10.1371/journal.pntd.0011615)
Supplement: S1 Fig — Single-nucleotide variations displayed along the aligned sequences of: A)ialB; B) gltA, consensus sequence includes only 782–1160 nucleotide sequences; C) rpoB, consensus sequence includes only 1451–2275 nucleotide sequences. (PDF) [file pntd.0011615.s001.pdf]

Supplementary material

	Nucleotide Region (nt position)	Consensus sequence	Nucleotide variation (nt position)
ialB (A)	1 – 60	ATG AAA AAA ATA TTA AAT TTA TTT GTA ATT TCT GCT TTC TTG AGT ATT TCC TCT GCA GCG	2 (50, 60)
	61 - 120	TTT GCT CAA AAT GTA AAA CCA GCT GCA ACG AAG CCT TCT GTT GCT ACA CTG CCG AAT GGT	1 (108)
	121 - 180	GCT TCT TCA TTA ACT GAG ACT TAC GGT TTG TGG AGC GTT AAC TGT GGT ATA CAG GAT GGG	2 (133, 138)
	181 - 240	AAC AAA ATT TGT ATT ATG CTT CGT CAG GAA GTT AAT GAG CAA GAT CGT GTT CTA TTG TCT	3 (183, 186, 234)
	241 - 300	ATG AGT GTT TCT CTT GAT GGG GAA GGT ACT GTA TCT GGC AAT TTG ACG ATT CCT TTT GGT	6 (245, 260, 267, 268, 269, 279)
	301 - 360	ATA TTG GTT TCT AAG CCT ATT CGT TTG CAT GTA GAT GAT TCG AAA TCT GTT ATT GAA AGC	5 (304, 312, 327, 342, 359)
	361 - 420	AAT GTT CGC ACT TGT GTG CCA GCA GGT TGT GTG GTT CCA ATA GTT TTT GAC AAA AAT TTT	1 (400)
	421 - 480	GTA GGG TCT TTA CGC GCT GGC AAG CAG TTG AAA TTA TCT ATG ACA GTT GCT GCT CCA GGT	4 (432,441, 448, 466)
	481 - 540	GAA CCA ACC TTG GAT AAT TTA TTT GTG CAA TTG GAT GGT TTC AGC AAC GCT CTT AAG CGT	5 (487, 489, 501, 515, 526)
	541 - 561	TTA ACT TCT TTG CAA AAA TAA	2 (545, 560)
gltA(B)	1 – 60	GG GGG CCA GCT CAT GGT GGA GCT AAT GAA GCA TGT CTA AAA ATG CTA CAA GAG ATA GGC	3 (5, 54, 60)
	61 - 120	TCT GTT AAA AAA ATT CCT CAA TTT ATT GCG CGT GCA AAA GAT AAA AAT GAT CCT TTT CGT	1 (79)
	121 - 180	CTT ATG GGC TTC GGC CAC AGA GTC TAC AAA AAT TAT GAT CCA CGT GCA AAG ATT ATG CAG	1 (171)
	181 - 240	AAA ACC TGC CAT GAA GTT TTA AAA GAG CTC AAC ATT CAA GAT AAC CCA CTT TTT GAT ATA	3 (202, 223, 232)
	241 - 300	GCG ATG GAG CTT GAG CAC ATC GCT CTG AAT GAT GAA TAT TTC ATT AAC AAA AAG CTC TAT	3 (255,285,297)
	301 - 360	CCT AAT GTC GAC TTC TAT TCT GGT ATT ACA TTA AAA GCT TTA GGA TTC CCT ACG GAA ATG	2 (315 – 354)
	361 - 380	TTT ACT GTT CTC TTC GCA TT	1 (375)
rpoB(C)	1 - 60	GT ATG GAG CGT GCG ATA AAG GAA CGC ATG TCT TCA GTT GAA ATT GAT ACT GTT ATG CCA	2 (15, 24)
	60 - 120	CAA GAT TTG ATT AAT GCG AAA CCA GCT GCG GCA GCT GTT CGC GAG TTT TTT GGG TCT TCG	4 (90, 102, 105, 114)
	121 - 180	CAA TTA TCG CAG TTT ATG GAT CAA ACT AAT CCA TTA TCA GAA ATT ACT CAT AAA CGT CGT	0
	181 - 240	CTT TCT GCT CTT GGG CCA GGT GGT TTA ACT CGT GAG CGT GCA GGT TTT GAA GTG CGC GAT	2 (195, 237)
	241 - 300	GTG CAT CCT ACG CAT TAT GGT CGT ATT TGT CCG ATT GAA ACG CCT GAA GGT CCA AAT ATT	3 (252, 273, 282)
	300 - 360	GGA TTG ATT AAT TCT TTA GCG ACT TTT GCG CGA GTT AAT AAA TAT GGT TTC ATT GAG AGT	4 (303, 321, 357, 360)
	361 - 420	CCA TAT CGC AAA ATT ACC GAT GGT AAA GTG ACG ACT GAG GTT GTT TAT CTT TCT GCT ATG	4 (378, 390, 393, 409)
	421 - 480	GAA GAA GCA AAA CAT TAT GTA GCT CAA GCC AAT TCT TCA CTA GAT TCA GAA GGA CGT TTC	1 (447)

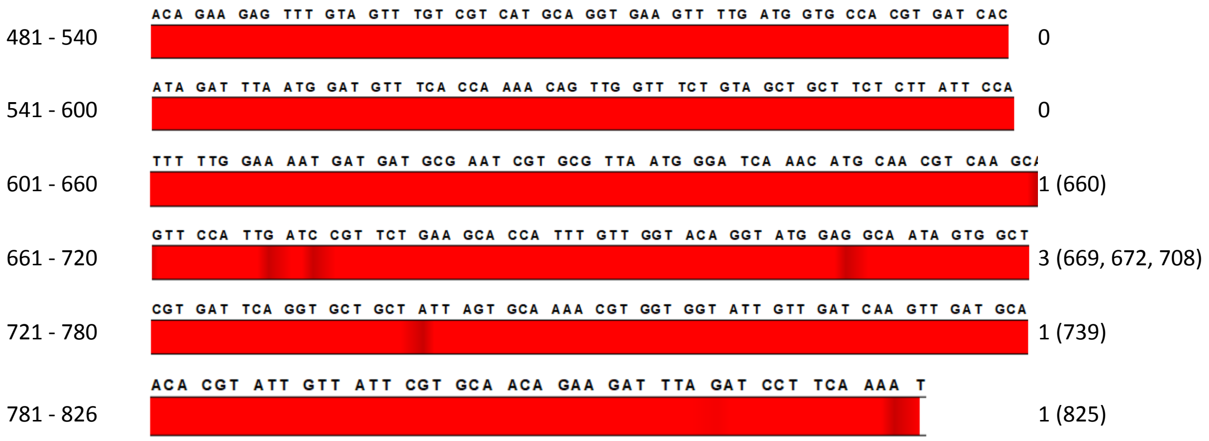

nt: nucleotide

S1 Fig. Single-nucleotide variations displayed along the aligned sequences of: A)*ial*B; B) *gltA,* consensus sequence includes only 782-1160 nucleotide sequences; C) *rpoB*, consensus sequence includes only 1451- 2275 nucleotide sequences
