# Supplementary material for: Single-nucleotide polymorphisms in ialB, gltA and rpoB genes of Bartonella bacilliformis isolated from patients in endemic Peruvian regions
Source: PLoS Negl Trop Dis. 2023 Oct 10;17(10):e0011615. doi: 10.1371/journal.pntd.0011615 (PMC10564245; doi:10.1371/journal.pntd.0011615)
Supplement: S2 Fig — The construction of the tree used the NJ method with 1,000 replicates. The Bootstrap values are displayed between the branches respectively. The tree was made in the MEGA 7.0 program. Red and green symbols highlight strains sequenced in the study. (PDF) [file pntd.0011615.s002.pdf]

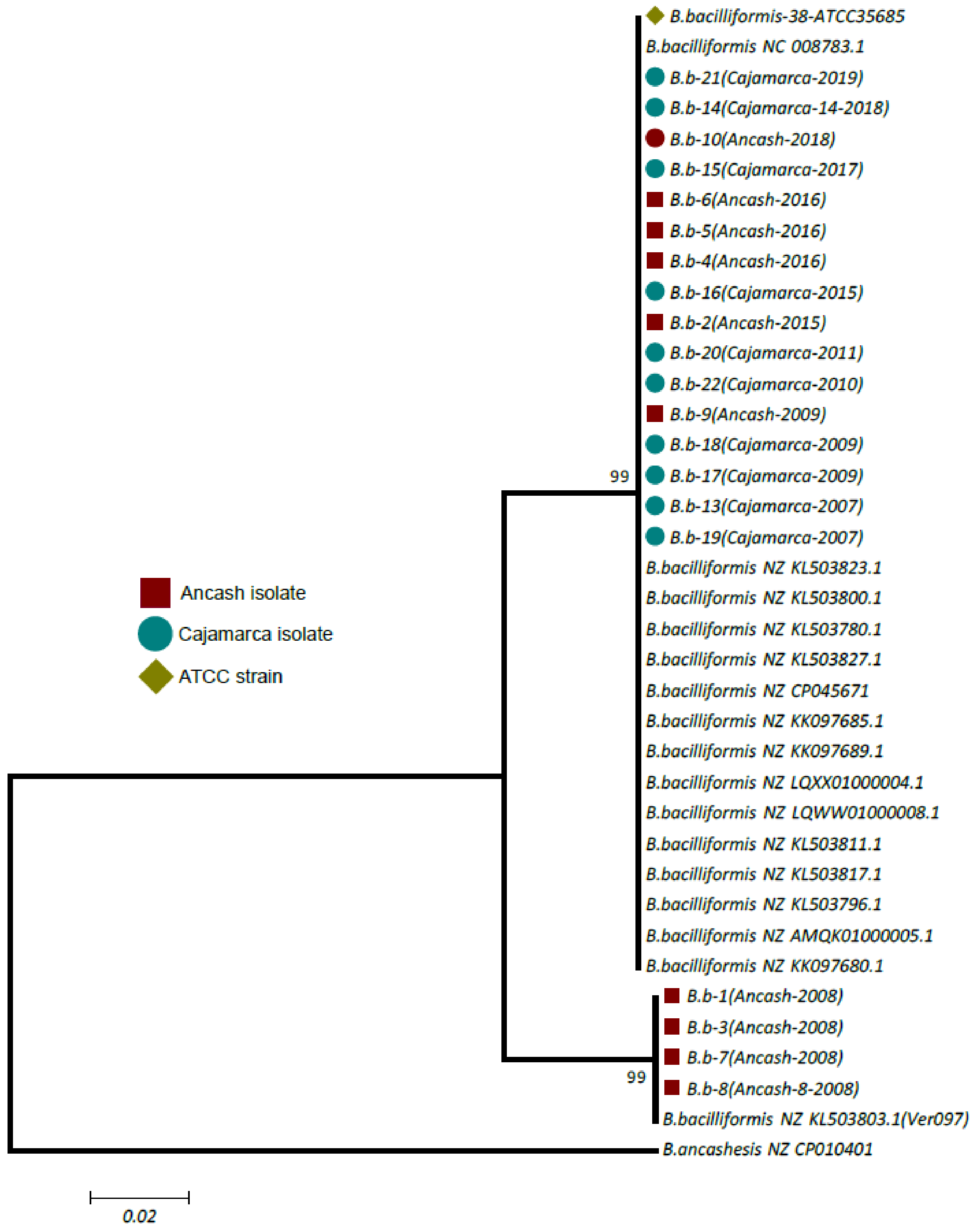

S2 Fig. Phylogenetic tree based on the *ialB* gene in 10 isolates from Cajamarca and 10 from Ancash and 17 genomes downloaded from the Genbank. The construction of the tree used the NJ method with 1,000 replicates. The Bootstrap values are displayed between the branches respectively. The tree was made in the MEGA 7.0 program. Red and green symbols highlight strains sequenced in the study.
