# Supplementary material for: Single-nucleotide polymorphisms in ialB, gltA and rpoB genes of Bartonella bacilliformis isolated from patients in endemic Peruvian regions
Source: PLoS Negl Trop Dis. 2023 Oct 10;17(10):e0011615. doi: 10.1371/journal.pntd.0011615 (PMC10564245; doi:10.1371/journal.pntd.0011615)
Supplement: S3 Fig — The construction of the tree used the NJ method with 1,000 replicates. Bootstrap values are shown between branches respectively. The tree was made in the MEGA 7.0 program. Red and green symbols highlight strains sequenced in the study. (PDF) [file pntd.0011615.s003.pdf]

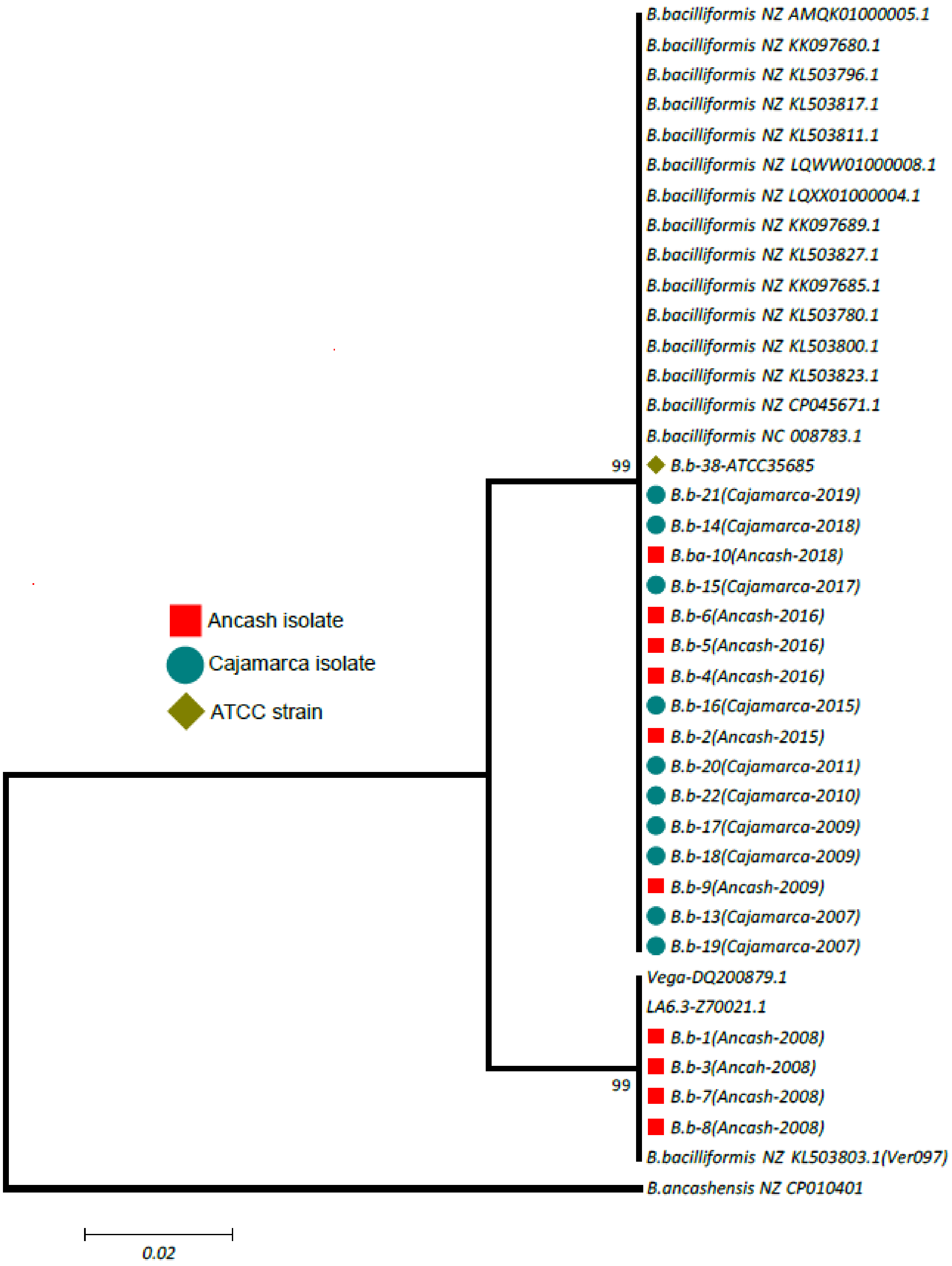

S3 Fig. Phylogenetic tree based on the *gltA* gene of 10 *B. bacilliformis* isolated in Cajamarca and 10 from Ancash; 17 genomes and 2 partial genes downloaded from the GenBank. The construction of the tree used the NJ method with 1,000 replicates. Bootstrap values are shown between branches respectively. The tree was made in the MEGA 7.0 program. Red and green symbols highlight strains sequenced in the study.
