# Supplementary material for: Single-nucleotide polymorphisms in ialB, gltA and rpoB genes of Bartonella bacilliformis isolated from patients in endemic Peruvian regions
Source: PLoS Negl Trop Dis. 2023 Oct 10;17(10):e0011615. doi: 10.1371/journal.pntd.0011615 (PMC10564245; doi:10.1371/journal.pntd.0011615)
Supplement: S4 Fig — For the construction of the tree, the NJ method with 1,000 replicates was used. Bootstrap values are shown between branches and evolutionary distances are calculated using Kimura’s 2-parameter method. The tree was made in the MEGA 7.0 program. Red and green symbols highlight strains sequenced in the study. (PDF) [file pntd.0011615.s004.pdf]

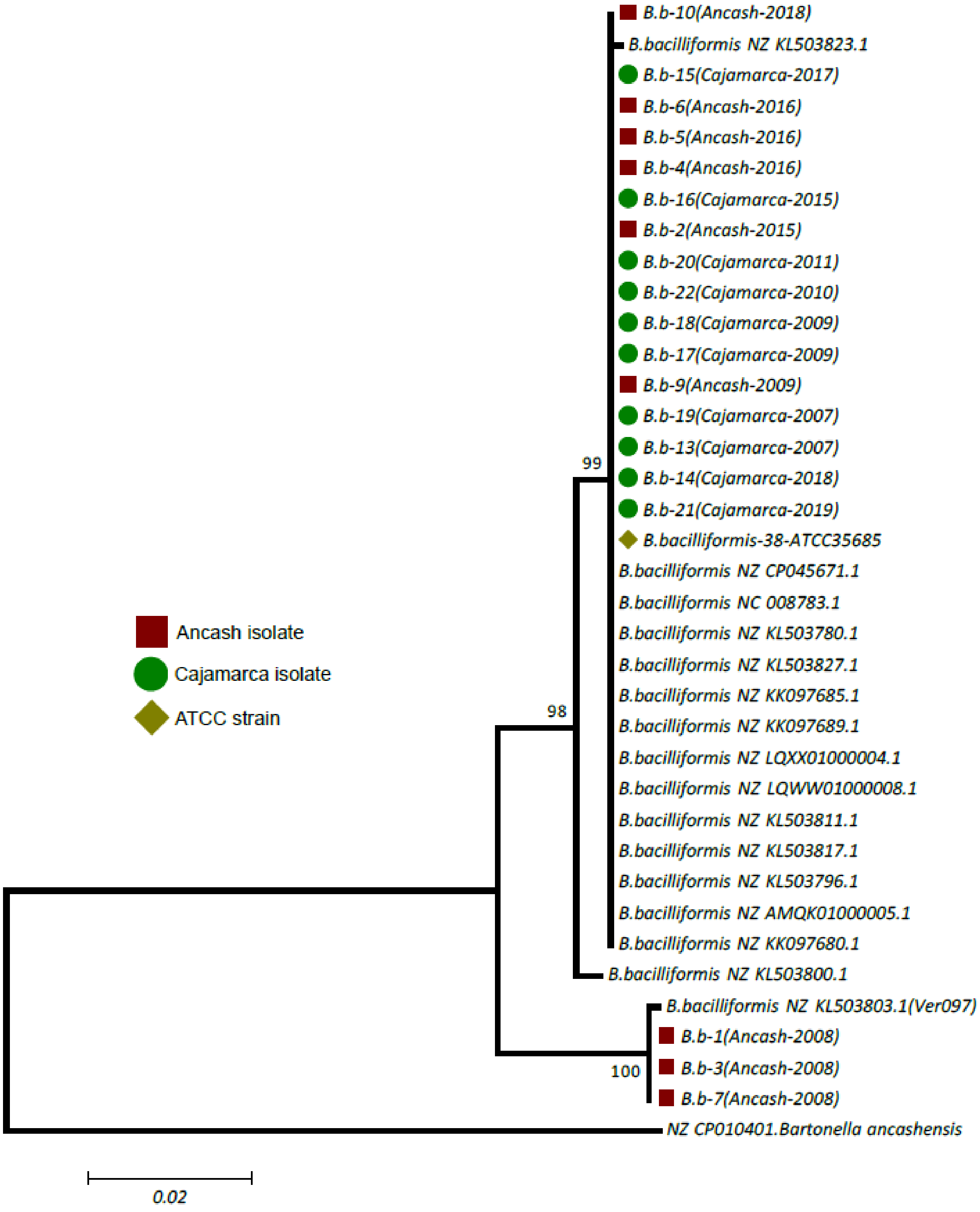

S4 Fig. Phylogenetic tree based on the *rpoB gene* from 10 *B. bacilliformis* isolates from Cajamarca and 10 from Ancash, and 17 genomes downloaded from the GenBank. For the construction of the tree, the NJ method with 1,000 replicates was used. Bootstrap values are shown between branches and evolutionary distances are calculated using Kimura's 2-parameter method. The tree was made in the MEGA 7.0 program. Red and green symbols highlight strains sequenced in the study.
